# Blocking Approach for Identification of Rare Variants in Family-Based Association Studies

**DOI:** 10.1371/journal.pone.0086126

**Published:** 2014-01-23

**Authors:** Asuman S. Turkmen, Shili Lin

**Affiliations:** 1 Statistics Department, The Ohio State University, Columbus, Ohio, United States of America; 2 Statistics Department, The Ohio State University, Newark, Ohio, United States of America; University of California, Irvine, United States of America

## Abstract

With the advent of next-generation sequencing technology, rare variant association analysis is increasingly being conducted to identify genetic variants associated with complex traits. In recent years, significant effort has been devoted to develop powerful statistical methods to test such associations for population-based designs. However, there has been relatively little development for family-based designs although family data have been shown to be more powerful to detect rare variants. This study introduces a blocking approach that extends two popular family-based common variant association tests to rare variants association studies. Several options are considered to partition a genomic region (gene) into “independent” blocks by which information from SNVs is aggregated within a block and an overall test statistic for the entire genomic region is calculated by combining information across these blocks. The proposed methodology allows different variants to have different directions (risk or protective) and specification of minor allele frequency threshold is not needed. We carried out a simulation to verify the validity of the method by showing that type I error is well under control when the underlying null hypothesis and the assumption of independence across blocks are satisfied. Further, data from the Genetic Analysis Workshop 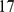 are utilized to illustrate the feasibility and performance of the proposed methodology in a realistic setting.

## Introduction

Genome-wide association studies (GWAS) have been extremely successful in identifying a bounty of common genetic variants linked to complex diseases and traits in the human population. While the identification of many novel variants associated with many traits has been a great accomplishment of GWAS, these genetic variants usually have small effect sizes and only account for a small proportion of the phenotypic variation. For example, height has been a well-known heritable quantitative trait with an estimated 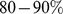 of the variation attributed to genetic factors [1], yet recent studies detected quite a number of loci that together only account for approximately 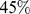 of the overall height variance [2]. Such observations have led to the hot topic of “missing heritability” [3], [4] and demonstrated the necessity of exploring other types of genetic variation that may account for unexplained heritability. With the ability to sequence the entire genome deeply, researchers have been looking beyond common sequence differences and interrogating rare single-nucleotide variations (rSNVs), i.e. variants of low minor allele frequency (MAF), that can contribute substantially to complex diseases. Therefore, many recent studies have focused on the possible contribution of rSNVs and they have hypothesized that some portion of this rare variation underlies much of the unexplained heritability of many complex traits [5].

Although assessing the role of rare variants in complex diseases is becoming increasingly feasible, detecting associations with rare variants still remains a challenging problem since rare variants are hard to pick up due to their low frequencies. Standard GWAS methods such as single-marker association tests are not appropriate strategies for detecting these low-frequency variants due to the fact that power diminishes with decreasing allele frequencies. As a result, a bevy of creative algorithms targeting rare variants have emerged. Such tests “collapse” information from rSNVs within a gene, a genomic region, a pathway, or some other defined properties. Without loss of generality and for ease of reference, we use genomic region when discussing such tests. Burden tests constitute a big portion of the existing literature [6]–[9]. These methods aim to maximize the power to detect causal variants by combining information across variants in a target genomic region which may be a gene or other functional unit. These tests provide a significant improvement over single-marker tests since each individual rare variant can make only a small contribution to the overall disease prevalence or trait variance, whereas their aggregate effect may constitute a significant attributable risk. While each of these burden tests differs in form, they all suffer from power loss when both protective and risk variants are present in the region of interest. Consequently, several methods that are robust to the direction and magnitude of the effects of causal variants have been proposed such as data-adaptive methods [10] and variance component based method (SKAT) that test the variance rather than the mean [11]. Recently, Lee et al [12] have proposed an unified approach that maximizes power by adaptively using the data to optimally combine the burden test and the non-burden SKAT test.

Investigators often use either population-based or family-based sampling designs to study the genetic basis of complex diseases. A population-based design samples affected and unaffected individuals who are unrelated, such as case-control samples. Almost all the methods proposed in the literature for detecting rare variant associations, including those discussed above, are for case-control studies. On the other hand, a family-based design treats a family as a sampling unit, which can be as simple as trios or as complex as large extended pedigrees with potentially multiple affected individuals per pedigree. For family studies, half of the offspring are expected to inherit a copy of the minor allele from a parent who has a copy of it. Therefore, variants that are rare in the general population could be quite common in certain families and are potentially more informative. Aggregation-based rare variant tests for analyzing family-data, however, are very limited and in fact only a handful have been proposed to date. Zhu et al. (2010) [13] propose a two-stage method that utilizes part of the data set to co-classify rare risk haplotypes either by unrelated-case or affected sibpair design, while the rest is used for association testing. It was demonstrated that the affected sibpair design has better power to co-classify rare risk haplotypes than the unrelated-case design due to the risk haplotype frequencies being more enriched in affected sibpairs than in affected cases. Similarly, Feng et al [14] developed a sibpair-based weighted sum statistic to detect both rare and common risk variants residing in a gene or a genomic region. Neither method models phenotypic or genetic correlations between related individuals and they cannot be used to analyze pedigree samples. Natural extensions of existing tests for population-based designs to family designs have also taken place in the literature. Two recent studies extended the idea of SKAT to family data [15], [16], but they only deal with quantitative traits. There is also an extension of a popular family-based association test (*FBAT*) to test for variants jointly over a region of interest [17]. Although the method can be used for both quantitative and qualitative traits, it relies on sometimes unrealistic assumption that all variants have effects in the same direction.

Due to low allele frequencies, it is hypothesized that rare variants do not exhibit strong linkage disequilibrium (LD) with either rare or common SNVs [18], [19]. Therefore, it has been argued in the last few years that rare rSNVs are independent, however, recent studies revealed that such an assumption is extremely liberal and can lead to significant inflation of type I error [20]. On the other hand, collapsing all rSNVs over a wide region and correcting for their dependence in an ad-hoc manner can lead to loss of power, since distant rSNVs are likely to exhibit independence, or at least such an assumption is not grossly violated. To balance the two extremes so that distant independence can be harnessed without sacrificing type I error, in this paper, we propose a blocking approach by assuming distant SNVs (i.e. SNVs between different blocks) are independent whereas dependency of SNVs within a block is taken into account. The proposed methodology does not require any assumptions about the directions of effect or the effect sizes of rare genetic variants in the region. Further, the approach can analyze all SNVs (rare or common) within a genomic region to avoid a preset threshold for rare variants. This is clearly an advantage over methods that require the specification of such a threshold as such methods are shown to be sensitive to the selection. Another important advantage of the proposed method inherited from family-based designs is its robustness to population stratification. The blocking approach is developed for two popular family-based common variant association tests for binary traits: *PDT* and *FBAT*. We evaluated the proposed methods based on simulation and application to the Genetic Analysis Workshop 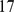 data. Comparisons with two *FBAT* extensions to rare variants by De et al. [17] were also carried out.

## Materials and Methods

### Family-based Association Tests - *PDT* and *FBAT* for a Single SNV

In this subsection, we first briefly describe two popular family-based tests, Pedigree Disequilibrium Test (*PDT*, [21]) and Family-Based Association Test (*FBAT* [22]), that are applicable to general pedigree data for testing for association in the presence of linkage. At the end of this subsection, we also describe two recent extensions of *FBAT* [17] for testing the aggregate effects of rSNVs. Our proposed rare blocking methods (*rbPDT* and *rbFBAT*) will be presented in the next subsection.

Let 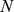 be the total number of pedigrees and 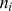 be the number of non-founders in the 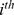 pedigree. Let 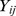 and 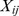 denote the trait value and the genotype score at the given SNV of the 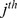 non-founder in the 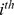 pedigree, respectively, where 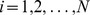; 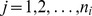. We assume that all 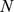 pedigrees in the data set are independent. In this study, we consider a binary trait where 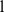 is case and 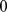 is control. At each SNV, genotype score is coded as 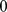, 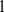 and 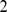 to represent 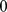, 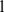 or 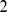 copies of minor allele.

The general test statistic for *FBAT* uses the covariance between the traits and the genotypes as a measure of association. Specifically,
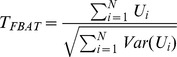
where 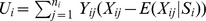 with 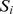 representing the sufficient statistic for parental genotypes, 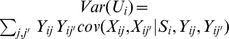 and the covariance in the formula is computed conditional on the traits and the sufficient statistic assuming the null hypothesis is true. Under the null hypothesis of no association, 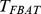 follows the standard normal distribution for large samples.

The *PDT* considers trios and discordant sibpairs in each pedigree. For a given SNV with two alleles 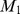 and 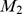, we define


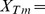
 (# 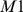 parental alleles transmitted)-(# 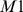 parental alleles not transmitted), for the 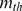 trio with an affected child and


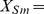
(# 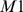 alleles in affected sib)-(# 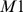 alleles in unaffected sib) for the 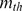 discordant sibpair. Then the *PDT* test statistic is
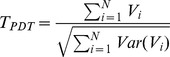
where 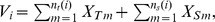 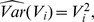 and 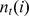 and 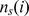 denote the number of informative nuclear families (consisting of one affected child and two parents) and discordant sibpairs (consisting of one affected sibling and one unaffected sibling), respectively, for the 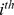 pedigree. Under the null hypothesis of no association, 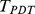 follows the standard normal distribution.

### Family-based Rare Association Tests -*FBAT* for Multiple SNVs

As in population-based approaches, single-SNV statistics may lack power to detect associations with rare variants. De et al [17] recently proposed a method that extends the *FBAT* statistic to test for rare variants by collapsing variants within a genomic region. More specifically, if there are 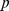 variants within the region, then 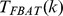 represents the value of the *FBAT* statistic for SNV 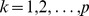 and the modified *FBAT* statistic for rare variants is defined as the sum of these 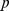 statistics. The standardized test statistic is obtained by dividing this sum by its standard deviation which is calculated using the correlation matrix estimate suggested by Rakovski et al. [23].

De et al. [17] considered analyzing either only rare variants using a fixed threshold or all variants within the region of interest using a weighting scheme. In the former, one can use a threshold value based on allele frequencies to identify a subset of variants as rare and consider the analysis on only that subset. Although 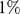 and 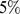 are the most conventional choices, setting the threshold too low may exclude some rare causal variants (those whose frequencies are above the threshold), yet setting it too high may include too many non-causal variants. Either way can potentially lead to loss of power. There may be further power loss if there are common causal variants. Alternatively, a weighting scheme based on allele frequencies is also proposed for joint analysis of common and rare variants. Throughout this paper, *fbatv0* and *fbatv1* denote the fixed threshold (using only rSNVs) and the allele frequency weighted (using all SNVs) approaches, respectively.

### *rbPDT* and *rbFBAT* - Blocking Approaches for Multiple SNVs

We propose a novel collapsing idea that can be incorporated into standard family-based tests *PDT* and *FBAT*. The idea is to balance the two extreme assumptions (all independence, or all dependence, of SNVs) that existing methods rely on by hypothesizing that distant SNVs are independent while dependency exists among nearby SNVs. In this approach, a genomic region is divided into blocks and SNVs within each block are aggregated to arrive at a statistic. Further aggregation is then performed across blocks but the block statistics are now assumed to be independent in the second layer of aggregation. The approach analyzes rare and common SNVs jointly and does not require a particular MAF threshold selection.

Suppose there are B blocks spanning the genomic region within which aggregate effect of the SNVs will be assessed. Details of the how to construct these blocks will be provided in the following subsection. Now consider block 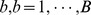, and assume that there are 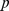 SNVs within the block. We consider a family-based test statistic (i.e. *FBAT* or the *PDT*) for the 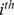 pedigree and 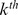 SNV as described in the previous section and denote it as 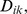 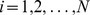; 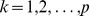. Then we define 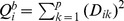 as the aggregate statistic for pedigree 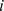 in block 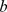. Note that by squaring the statistic for each SNV, one can prevent effects of deleterious and protective variants within the block to cancel out each other. Under the null hypothesis of no association, 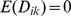 implying that 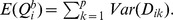 The pooled variance of the 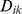
's provides an estimate for 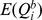, and is denoted as 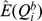. We define 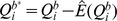, then
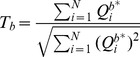
denotes the standardized statistic utilizing information from all SNVs within block 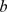 for all pedigrees, which follows a standard normal distribution approximately assuming 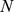 is sufficiently large. We then sum over information from all 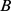 blocks to arrive at the following overall test statistic:



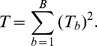



Assuming the 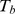
's are independent, the statistic 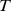 follows a chi-square distribution with 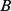 degrees of freedom. If the 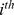 pedigree *PDT* statistic (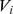) for the 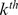 SNV is used as 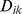, then the resulting method is called rare-blocking *PDT*, or *rbPDT*, while the one that uses the 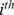 pedigree *FBAT* statistic (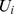) for the 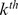 SNV is termed *rbFBAT*.

### Blocking Schemes

Sliding-window approach that considers several neighboring SNVs together within a window frame is a popular strategy for collapsing methods. We borrow this simple idea by arranging all SNVs of interest into “independent” blocks. Each block can be viewed as a “window”, while the window (block) size is determined by either the number of base-pairs (bp; the typical sense of a window) or by the number of SNVs (a novel concept). Determination of a good window size in either definition is an important but difficult problem as its choice can be influenced by factors such as LD patterns and MAF of the causal SNVs. Since it is not feasible to investigate the relationship between optimal window size and these factors analytically, we consider several reasonable options and evaluate how blocking schemes may affect the performance of *rbPDT* and *rbFBAT* empirically.

Although multiple genes (in the thousands) are being considered in this study, for simplicity, each blocking scheme with multiple window sizes are applied to all genes to gain an understanding of the overall performance so that general recommendation can be made. For example, if the window size is set to be 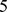 kb, then all genes are partitioned into 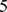 kb blocks. Note that this blocking scheme may result in different number of blocks for different genes since gene lengths vary. Another possible approach is to specify the number of blocks and divide each gene (large or small) into that many blocks. Unlike the former approach, this may result in blocks of difference sizes in different genes, also depending on gene lengths. Similar phenomenon applies to the scenario when “window size” is defined as the number of SNVs it contains. One way of constructing such blocks is to fix the number of SNVs in each block (e.g. 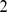 SNVs per block). Note that this will result in different number of blocks depending on the gene size and SNV density. Another way is to fix the number of blocks and partition all SNVs into the specified number of blocks so that each block has the same number of SNVs. This may result in different sizes (i.e. different number of SNVs in each block) across genes. These ideas lead to the following four options that are employed in the analyses carried out in this paper. The numbers in parentheses specify the number of blocks or window size (which can either be in bp or in number of SNVs depending on which definition of window size is being used).

**Option 1**: Each gene is divided into blocks of equal length in bp (e.g. 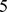 kb);

**Option 2**: Each gene is divided into fixed number of blocks of equal length in bp (e.g. 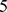 blocks);

**Option 3**: Each gene is divided into blocks containing equal number of SNVs (e.g. 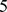 SNVs per block);

**Option 4**: Each gene is divided into fixed number of blocks containing equal number of SNVs (e.g. 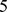 blocks).

To further clarify and deal with unconventional situations, we note that Options 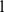 and 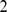 use bp to define a window size whereas the window size used in Options 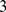 and 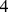 is number of SNVs. In each case, one can either fix the number of blocks or the window size when constructing blocks. If the gene length (either in bp or in number of SNVs) is less than or equal to a specified window size, then only one block is formed for Options 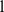 and 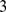. When Option 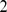 is employed, only blocks containing at least one SNV are kept for further analysis since some blocks may not contain any SNVs. If the number of SNVs within a gene is less than the specified number of blocks under Option 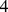, it constructs blocks to have one SNV in each block. That is, the number of blocks for that gene is set to be equal to the number of SNVs within the gene. Therefore, for fixed number of blocks options (both 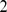 and 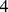), it is possible that some genes are divided into fewer number of blocks than the pre-specified number. A comprehensive comparison of these blocking strategies will be provided in the Results section.

### Genetic Analysis Workshop 17 Data

The Genetic Analysis Workshop 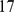 (GAW 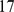) mini-exome data contain genotypes for 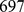 unrelated individuals on 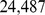 variants (in 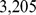 genes) from the 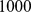 Genomes Project. The workshop also distributed data on 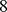 extended pedigrees composed of 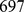 individuals. Of the 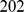 founders, their genotypes were assigned based on a random sample from the 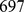 unrelated individuals, which turned out to include 12 CEPH (European-descent residents of Utah), 18 Denver Chinese, 19 Han Chinese, 28 Japanese, 50 Luhya, 66 Tuscan, and 9 Yoruban individuals. As such, there exists population admixture. These genotypes are then dropped down to form the genotypes of non-founders, leading to some of the rare variants (such as private variants that only appear once in the 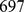 unrelated individuals) being enriched in the family sample. Based on the fixed genotype data, 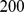 sets (replicates) of binary disease phenotypes were simulated according to a model that portraits 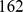 causal SNVs in 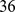 genes, with approximately 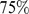 of these causal variants having MAF less than or equal to 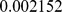. The first and third quartiles of the causal variant percentages within 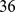 causal genes were 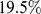 and 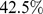, respectively. A more complete description of the GAW 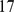 data is provided by Almasy et al. (2011) [24].

Our numerical analysis starts with a simulation study designed to evaluate type I error and power for *rbPDT*, *rbFBAT*, *fbatv0* and *fbatv1* under different scenarios. In this simulation, we generated genotype and phenotype data utilizing the same pedigree structures as in the GAW 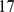 data. After the simulation demonstrating the validity of the proposed methodologies, we performed two sets of analyses of the GAW 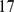 data. In the first set of analyses, we investigate how blocking schemes with various window sizes and block numbers may affect the outcomes of *rbPDT* and *rbFBAT* by using only the first replicate of simulated phenotypes. This analysis also provides an evaluation of the effectiveness of *rbPDT* and *rbFBAT* for detecting rare variants using a realistic data set. The second set of analyses is carried out to compare the performances of *rbPDT* and *rbFBAT* with those of *fbatv0* and *fbatv1* using phenotype data from all 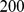 replicates. For each gene, we set its length to equal the difference between the largest and the smallest base-pair locations of SNVs contained in the gene. With this definition, the majority of these 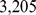 genes have length less than 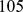 kb with 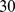 or fewer SNVs.

## Results

### Simulation Study: Type I Error and Power

To verify the validity of the proposed blocking methods and to compare them with recently proposed state-of-art methods for family-based studies in a controlled setting, we perform a simulation study in which the family structure from GAW 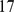 data is replicated 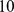 times to create 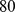 families so that the sample size is sufficiently large to draw definitive conclusions, especially on the type I error. We simulated 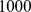 sets (replicates) of genotype data for the founders, which consists of 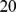 SNVs modeled after the *LPL* gene provided in the GAW 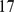 data and with linkage disequilibrium (LD) setting that utilizes a block diagonal correlation matrix assuming there are 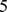 independent blocks. Within each block containing 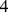 SNVs, LD correlation between farther SNVs decays following an autoregressive model with 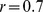. Under this setting, the optimal blocking is the one that divides the SNVs in groups of 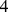 SNVs, i.e. Option 4 of our blocking schemes with 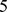 blocks, as it preserves the independence assumption across blocks. Haplotypes for unrelated founders with desired MAF and LD structure are simulated using the same procedure as in HapSim [25], then we passed down the haplotypes to the remaining individuals assuming no recombination. For each set of genotypes, the binary disease status for each individual was simulated using the model
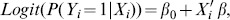
(1)where 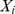 and 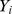 denote the genotype and trait values for the 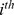 individual. Type I error was calculated using the model with 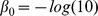 and 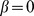, while 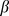 is set to be either 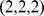, 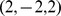, 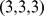 or 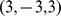 for three (as in the *LPL* gene) randomly selected causal variants. This process is repeated 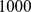 times to create 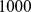 data sets to investigate the size and power under settings with different effect size and directions. Results for *fbatv1* using allele frequency weights, *fbatv0* with three different MAF thresholds (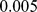, 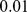, and 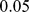), *rbPDT* and *rbFBAT* with one specification for each option (i.e., 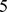 kb for Option 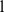, 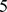 SNVs for Option 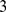, and 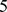 blocks for Options 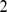 and 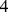) are reported in Table 1 with the significance 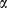-level set to be 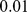. We also include the one-block (i.e. no blocking) option as a comparison. Since the actual type I error rates are not comparable across different methods, we adjusted the critical value so that all tests have the same 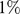 error rate to compute the power reported in Table 1.

**Table 1:** Type 1 error and power results for the simulation based on a data set with 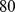 families.

		Type I	Power			
Method	Option	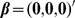	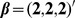	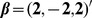	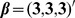	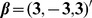
rbPDT	Option 1	0.014	0.538	0.385	0.896	0.868
	Option 2	0.005	0.108	0.107	0.638	0.615
	Option 3	0.017	0.138	0.116	0.717	0.687
	Option 4	0.007	0.366	0.245	0.802	0.765
	1 Block	0.037	0.487	0.328	0.544	0.579
rbFBAT	Option 1	0.132	0.475	0.269	0.718	0.628
	Option 2	0.017	0.495	0.452	0.862	0.788
	Option 3	0.046	0.488	0. 344	0.834	0.756
	Option 4	0.011	0.758	0.542	0.945	0.915
	1 Block	0.170	0.250	0.130	0.486	0.412
FBAT	v1 (weighted)	0.008	0.155	0.027	0.837	0.555
	v0 (0.005)	0.008	0.098	0.024	0.561	0.379
	v0 (0.01)	0.007	0.091	0.028	0.623	0.413
	v0 (0.05)	0.005	0.170	0.040	0.767	0.533

The nominal 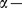
level is set to be 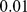. The between-block independence structure holds under Option 4.

The results indicate that type I error is maintained with a nominal 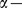
level of 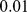 when the between-block-independence assumption is satisfied (Table 1, Option 4 for both *rbPDT* and *rbFBAT*). When the assumption of independence between blocks is violated (as in Options 1–3), there can be some inflation of type I error (especially Option 1 under *rbFBAT*). However compared to the one-block approach, the type I error is better under controlled. On the other hand, The *fbatv0* with all MAF thresholds and *fbatv1* are a bit conservative. As expected, power increases with effect sizes in all methods. When SNVs have opposite effect directions, substantial power loss is observed for *fbatv0* and *fbatv1*, while the power based on blocking approaches remains comparable with the power of the same direction effect sizes.

### Analysis of GAW 17 Data

#### Analysis I

In the first analysis, different blocking options with various specifications are compared utilizing the disease status from the first replicate. Specifically, two fixed window size and two fixed number of blocks are used with varying window size and number of blocks: Option 1 (2,3,4,5 or 6 kb), Option 2 (2, 3, 5, 7, 10 blocks), Option 3 ((2, 3, 5, 7, 10 SNVs) and Option 4 (2, 3, 5, 7, 10 blocks).

**Figure 1:**
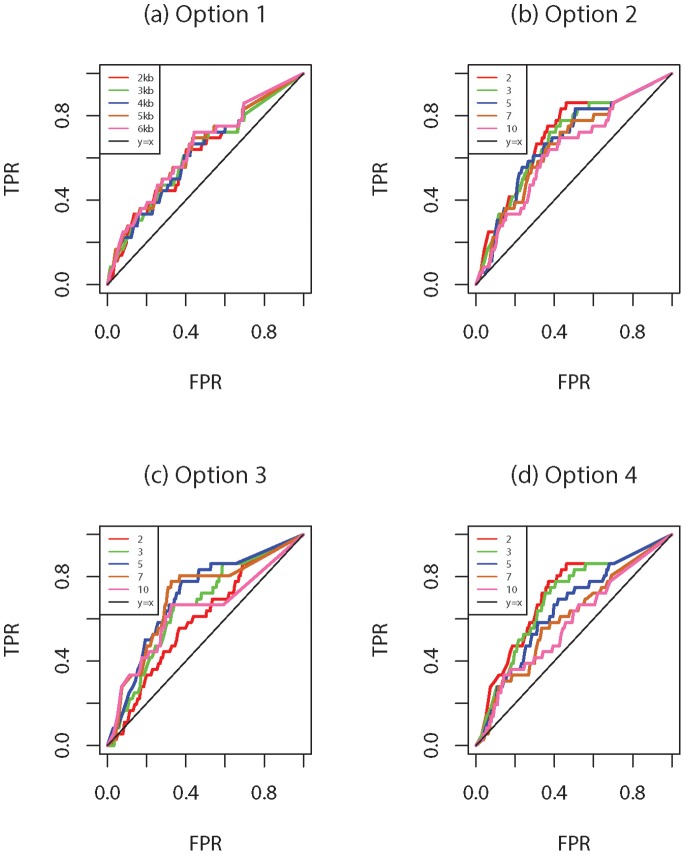
*rbPDT* results using different blocking options: (a) Option 1 with block size 2, 3, 4, 5, or 6 kb; (b) Option 2 with number of blocks 2, 3, 5, 7, or 10; (c) Option 3 with blocks including 2, 3, 5, 7, or 10 SNVs; (d) Option 4 with number of blocks 2, 3, 5, 7, or 10.

**Figure 2:**
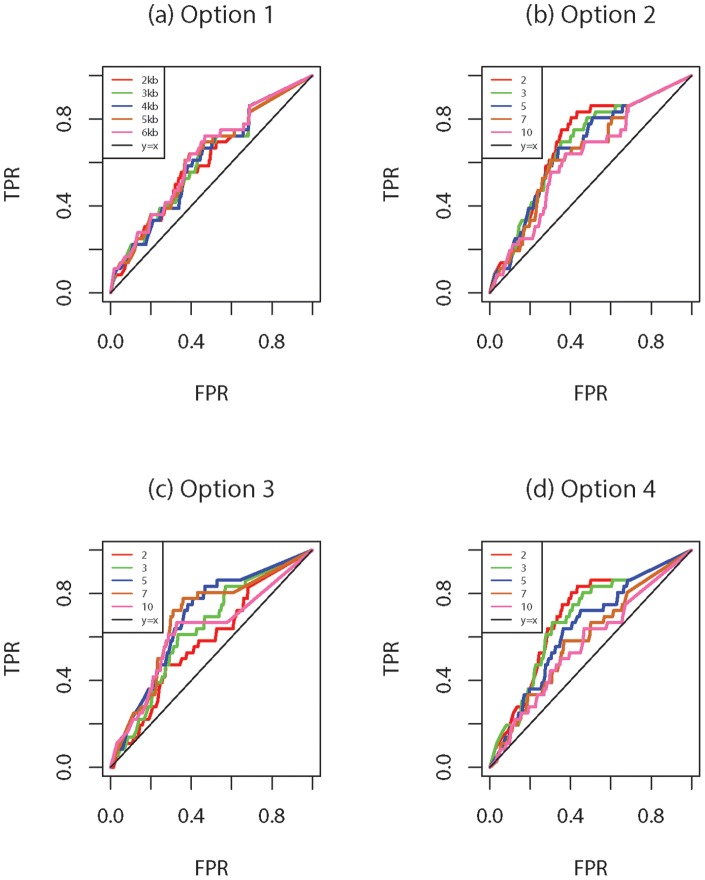
*rbFBAT* results using different blocking options: (a) Option 1 with block size 2, 3, 4, 5, or 6 kb; (b) Option 2 with number of blocks 2, 3, 5, 7, or 10; (c) Option 3 with blocks including 2, 3, 5, 7, or 10 SNVs; (b) Option 4 with number of blocks 2, 3, 5, 7, or 10.

The results are provided in Figures 1 and 2. Here, true positive rate (TPR; power) is defined as the proportion of all causal genes that are found to be significant, i.e. (total number of true positives)/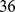, whereas the false positive rate (FPR; type I error) is the proportion of all non-causal genes that are found to be significant, i.e. (total number of false positives)/(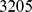-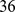). Figure 1 show the receiver operating characteristic (ROC) curves obtained by *rbPDT* for the different options with different size or number of blocks selections. The 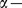
level significance is between 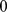 and 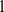 with 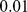 increments. Different choices of window size result in more fluctuations for blocking using the fixed number of SNVs (Option 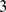) than the blocking using fixed number of bp's (Option 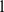). One possible explanation for this might be that since most of the gene lengths are 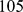 kb or less, dividing them into 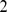, 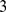, 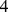, 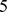, or 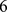 kb blocks yields similar partitions containing the same set of SNVs especially if SNVs are densely distributed along the gene. On the other hand, splitting SNVs of a gene into blocks with different number of SNVs may result in quite altered separations. Two fixed number of blocks approaches, Options 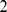 and 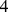, produce more variations compared to their block size counterparts, Options 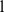 and 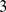. This is not surprising since different number of blocks are more likely to create variations in building blocks especially when the number of SNVs within the gene or gene length is large. Nevertheless, despite these variations, all blocking methods with different size selections yielded very close power and type I errors especially when FPR is small (lower-left corner of Figure 1). Results based on *rbFBAT* are illustrated by ROC curves in Figure 2. Like *rbPDT*, *rbFBAT* results are more stable for Option 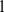 than Option 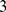 while both yield less variation than their counterparts with a fixed number of blocks (Option 1 vs. Option 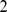, and Option 3 vs. Option 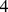). Performances of *rbPDT* and *rbFBAT* are very comparable as shown by the figures. Overall, Figures 1 and 2 indicate that *rbPDT* and *rbFBAT* are at par and blocking scheme selection might have an effect on the results especially depending on the distribution of gene lengths and number of SNVs within the genes.

**Figure 3:**
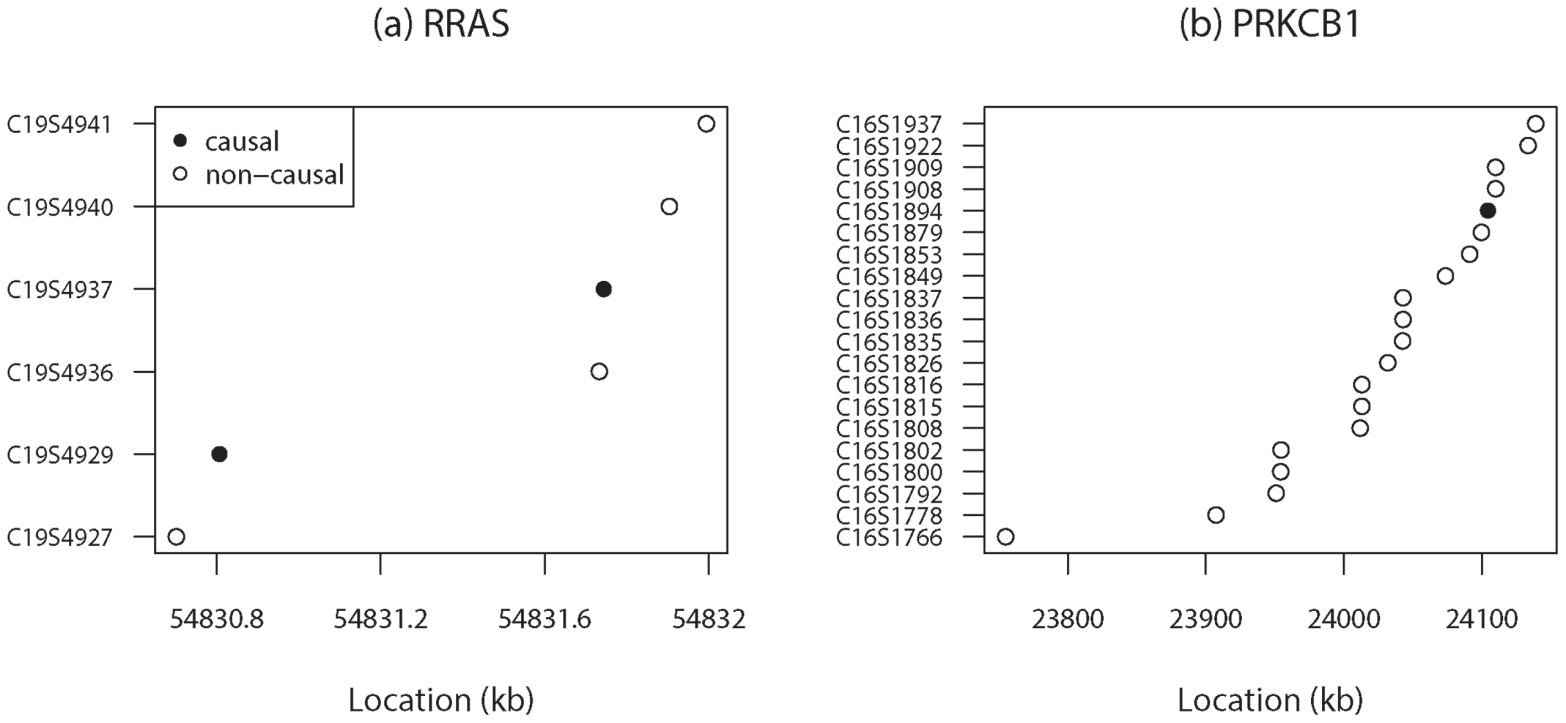
SNV base-pair locations for two-causal genes: (a) RRAS: the average distance between two successive SNVs is 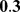 kb with a maximum distance of 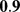 kb; (b) PRKCB1: the average distance between two SNVs is 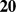 kb with a maximum distance of 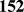 kb.

To further investigate the effect of blocking, we select 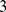 causal genes (*INSIG1*, *LPL*, *PIK3C2B*) containing small, moderate and large number of SNVs (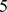, 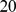 and 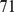) and 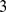 causal genes (*RRAS*, *HIF3A*, *PRKCB1*) with small, moderate and large lengths (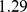, 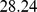, and 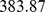 kb). The p-values for these genes obtained from *rbPDT* are listed in Table 2 for all options with different size selections. We omit results from *rbFBAT* for brevity since the inferences are quite similar. It can be seen that Option 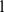 results for different window sizes vary less compared to Option 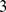 results, consistent with the observations made from Figures 1 and 2. We believe that this is due, in part, to the fact that Option 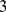 produces varying number of blocks and thus containing different sets of SNVs yielding a range of p-values. In general, it appears that for small to moderate size genes containing small to moderate number of SNVs, the p-value tends to get larger as the number of blocks increase, leading to reduction in power. Therefore, for such genes, blocking schemes leading to a small number of blocks might be a better option. This is especially true if most of the causal SNVs are rare and combining as many as possible can strengthen the signals. These inferences are particularly true for genes *INSIG1*, *LPL*, *RRAS*. Although *HIF3A* is also a moderately large gene with moderate number of SNVs, it is not true that small number of blocks yield optimal results. When we look at this gene more closely, we see that SNVs are sparsely distributed along the gene, so setting a small number of blocks results in including distant SNVs in the same block. Since, in general, it is more likely to observe sparsely located SNVs for larger genes, such as *PIK3C2B*, options with smaller number of blocks may not work as in small genes. In such cases, one should consider an option that will produce a reasonable number of blocks such that distant SNVs are not combined into same block yet close SNVs are not separated. This finding shows that, beside the gene length and SNV number within the gene, how closely SNVs are spaced on the gene is another important factor that can create variations for different size selections. In Figure 3, the base pair locations of the 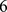 SNVs in the *RRAS* gene (a small one) and 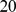 SNVs in the *PRKCB1* gene (a larger one) are plotted. For the *RRAS* gene, although there are two visible clusters, as can be seen in Figure 3(a), they are not far separated since the gene is very small. At the same time, since both of the causal SNVs are very rare (MAF = 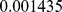), constructing one block (including all 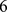 SNVs) provides more power than the two blocks scheme does. On the other hand, SNVs located in *PRKCB1* are very sparse. Obviously, very large number of blocks (see Option 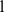 results for *PRKCB1*) is not a good approach due to its large degrees of freedom. Very small number of blocks may not be the right choice either. For instance, the smallest p-value of 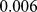 is obtained by Option 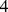 with three blocks while the p-value for two block approach is 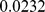. The latter leads to the observation that there may be power loss if distant non-causal SNVs are grouped together with causal ones into the same block.

**Table 2:** List of p-values for six causal genes for different options with a variety of partitioning choices.

Option	Gene	INSIG1	LPL	PIK3C2B	RRAS	HIF3A	PRKCB1
	#SNVs	5	20	71	6	21	20
	Width(kb)	6.62	14.02	28.49	1.29	28.24	383.77
1	2 kb	0.211(2)	0.076(7)	0.022(18)	0.044(1)	0.008(6)	0.266(14)
	3 kb	0.211(2)	0.064(5)	0.008(14)	0.044(1)	0.008(6)	0.266(14)
	4 kb	0.211(2)	0.033(4)	0.005(12)	0.044(1)	0.008(6)	0.266(14)
	5 kb	0.211(2)	0.019(3)	0.006(9)	0.044(1)	0.004(5)	0.208(13)
	6 kb	0.211(2)	0.019(3)	0.003(8)	0.044(1)	0.003(5)	0.208(13)
2	2 blocks	0.211(2)	0.018(2)	0.011(2)	0.131(2)	0.011(2)	0.028(2)
	3 blocks	0.211(2)	0.019(3)	0.007(3)	0.131(2)	0.006(3)	0.024(3)
	5 blocks	0.211(2)	0.076(5)	0.001(5)	0.112(3)	0.005(4)	0.062(5)
	7 blocks	0.374(3)	0.069(6)	0.003(7)	0.112(3)	0.008(6)	0.019(6)
	10 blocks	0.374(3)	0.111(7)	0.002(10)	0.199(4)	0.008(6)	0.025(7)
3	2 SNVs	0.374(3)	0.114(10)	0.198(36)	0.112(3)	0.058(11)	0.072(10)
	3 SNVs	0.211(2)	0.061(7)	0.033(24)	0.050(2)	0.022(7)	0.027(7)
	5 SNVs	0.078(1)	0.058(4)	0.002(15)	0.131(2)	0.010(5)	0.009(4)
	7 SNVs	0.078(1)	0.036(3)	0.001(11)	0.044(1)	0.014(3)	0.006(3)
	10 SNVs	0.078(1)	0.018(2)	0.003(8)	0.044(1)	0.030(3)	0.023(2)
4	2 blocks	0.211(2)	0.018(2)	0.011(2)	0.050(2)	0.011(2)	0.023(2)
	3 blocks	0.374(3)	0.036(3)	0.006(3)	0.112(3)	0.014(3)	0.006(3)
	5 blocks	0.682(5)	0.052(5)	0.001(5)	0.220(5)	0.020(5)	0.016(5)
	7 blocks	0.682(5)	0.061(7)	0.002(7)	0.320(6)	0.022(7)	0.027(7)
	10 blocks	0.682(5)	0.114(10)	0.000(10)	0.320(6)	0.038(10)	0.072(10)

Number of the blocks (degrees of freedom) are provided in the parentheses.

#### Analysis II

**Figure 4:**
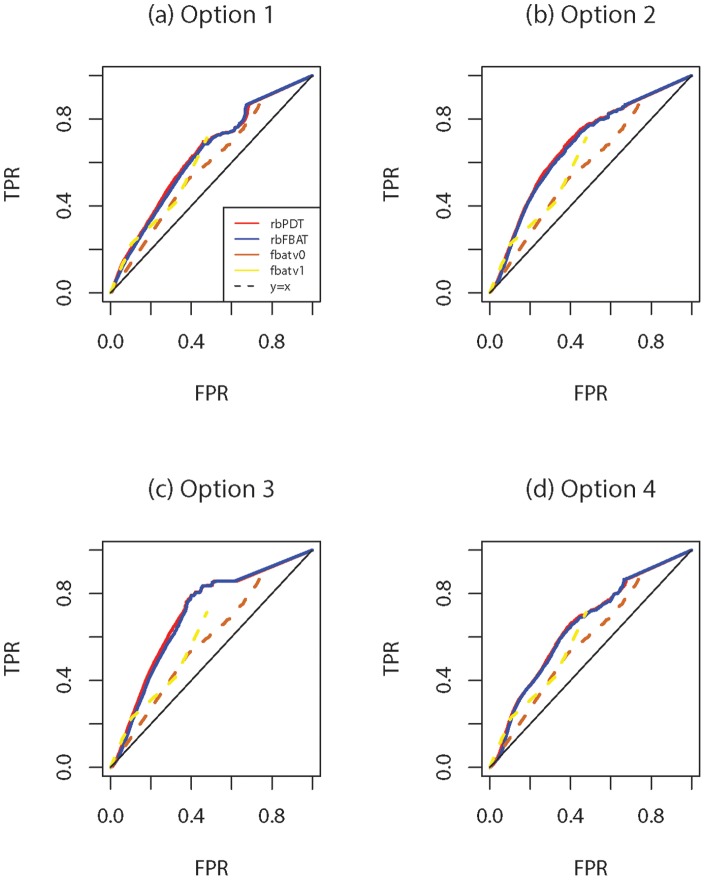
200 replicate results: ROC curves for *fbatv0*, *fbatv1*, (a) *rbPDT* and *rbFBAT* with Option 1; (b) *rbPDT* and *rbFBAT* with Option 2; (c) *rbPDT*and *rbFBAT* with Option 3; (d) *rbPDT* and *rbFBAT* with Option 4.

**Figure 5:**
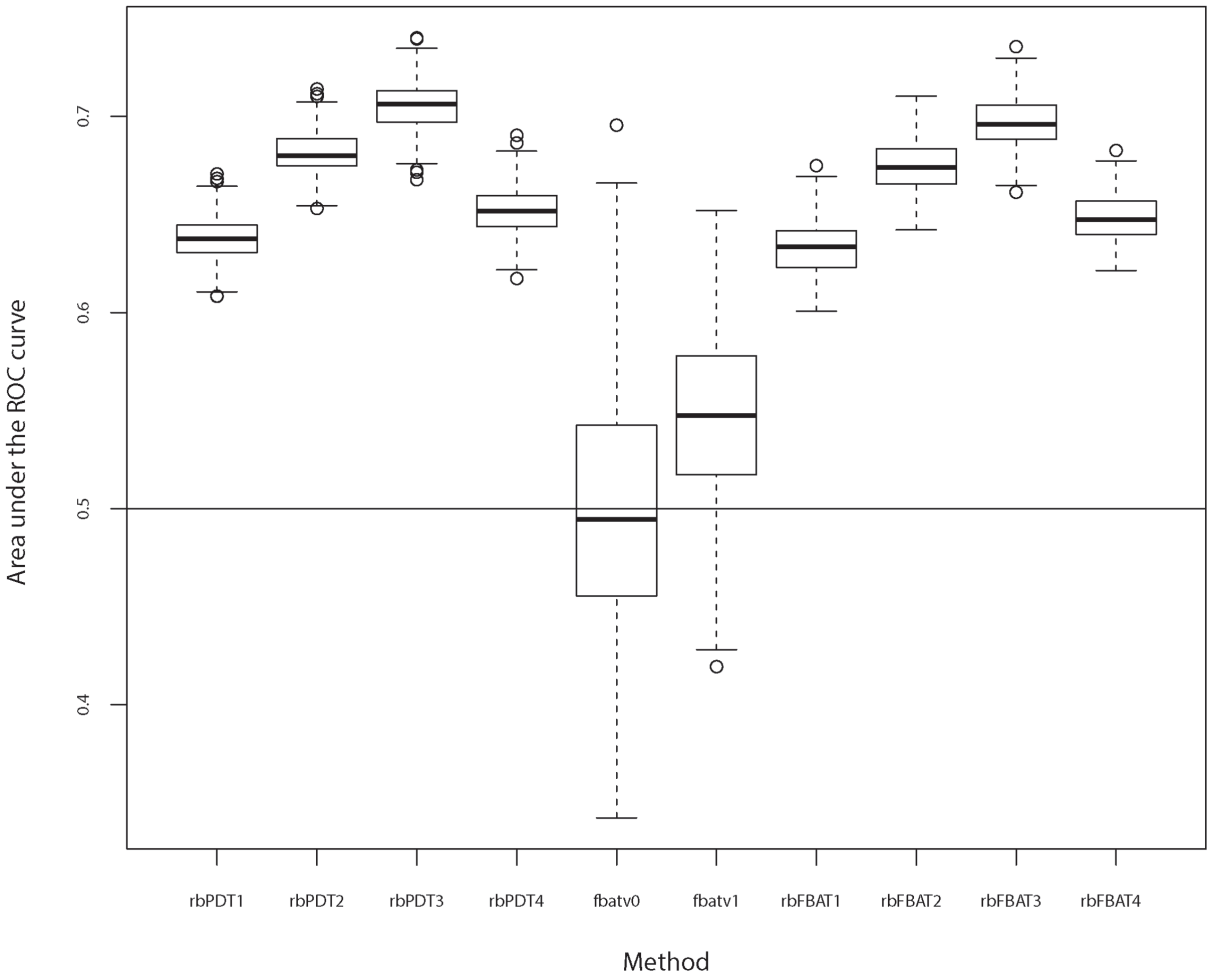
200 replicate results: Boxplots of the area under the ROC curves for 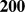 replicates using *fbatv0*, *fbatv1*, and *rbPDT* and *rbFBAT* with four options.

In the second set of analysis, we compare the performance of *rbPDT* and *rbFBAT* with two recently proposed methods, *fbatv0* and *fbatv1* [17]. We chose *fbatv0* and *fbatv1* since they are also extensions of *FBAT* and software implementations are available (beta version of *FBAT* v2.0.4). We set the MAF threshold for *fbatv0* as 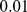 and use the weighting scheme based on allele frequencies as described in [17] for the 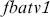. We utilized all 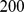 replicates to construct ROC curves for each method. For *rbPDT* and *rbFBAT*, only one representative from each option (i.e., 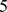 kb for Option 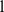, 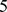 SNVs for Option 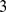 and 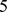 blocks for Option 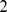 and 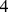) is used. Power is defined as the average percentage of all causal genes that are found to be significant, i.e. (total number of true positives)/(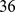
*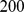), whereas type I error is the average percentage of all non-causal genes that are found to be significant, i.e. (total number of false positives)/[(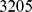-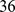)*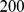]. ROC curves for *fbatv0*, *fbatv1*, *rbFBAT*, *rbPDT* for options 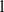, 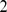, 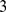 and 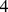 are given in Figure 4 (a), (b), (c) and (d) respectively. Overall, *fbatv1* is better than *fbatv0*, consistent with the original finding by De et al. [17] in that fixed threshold approach *fbatv0* is highly dependent on the choice of the MAF threshold while weighted no threshold approach *fbatv1* avoids such choice problem and outperforms *fbatv0*. On the other hand, both blocking methods, *rbFBAT* and *rbPDT*, perform similarly and are better than the two fbat versions. These findings are further substantiated by the boxplots shown in Figure 5, in which area under the ROC curves (AUC) for 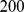 replicates are shown for each of the method and blocking options. It can be seen that *fbatv0* and *fbatv1* are clearly outperformed by *rbPDT* and *rbFBAT* with any of the four blocking options. Further, *rbPDT* and *rbFBAT* continue to be similar evaluated by AUC.

## Discussion

Family-based designs have important advantages over population-based methods, especially in their robustness to population stratification. Motivated by this, we extend two popular family-based association approaches originally proposed for common variants, *PDT* and *FBAT*, to test for association with rare variants. These approaches (*rbPDT* and *rbFBAT*) are based on splitting a given genomic region (e.g. gene) into blocks under the assumption that SNVs between different blocks are independent while dependency among SNVs within a block is being taken into consideration. A statistic for each block is calculated using the SNVs located within the block and statistics across these blocks are combined to arrive at an overall test statistic for the genomic region of interest. An inherent assumption for our test statistic to be valid is that the statistics across blocks that summarize information from multiple SNVs are independent. However, dependency among SNVs within a block is allowed and accounted for. As such, the proposed methodology may be viewed as a compromise between methods that assume independence of all rare SNVs and those that depict dependency of all variants within a region. This compromise can lead to increase in power by “creating independent data” while holding the type I error rate at base since it still accounts for dependency in a proper fashion. As an further advantage, our methods do not require an MAF threshold and can analyze rare and common variants jointly. We show that, based on ROC (AUC) evaluations, *rbPDT* and *rbFBAT* perform similarly and reasonably well compared to two recently proposed family-based methods, *fbatv0* and *fbatv1*, for detecting rare variants. For a fixed significance level (e.g. 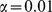), *rbPDT* and *rbFBAT* may outperform each other under different blocking options, but they consistently outperform *fbatv0* and *fbatv1*. More importantly the empirical type I error rates of both *rbPDT* and *rbFBAT* are close to the nominal level specified when the underlying assumptions for the validity of the asymptotics are satisfied.

**Figure 6:**
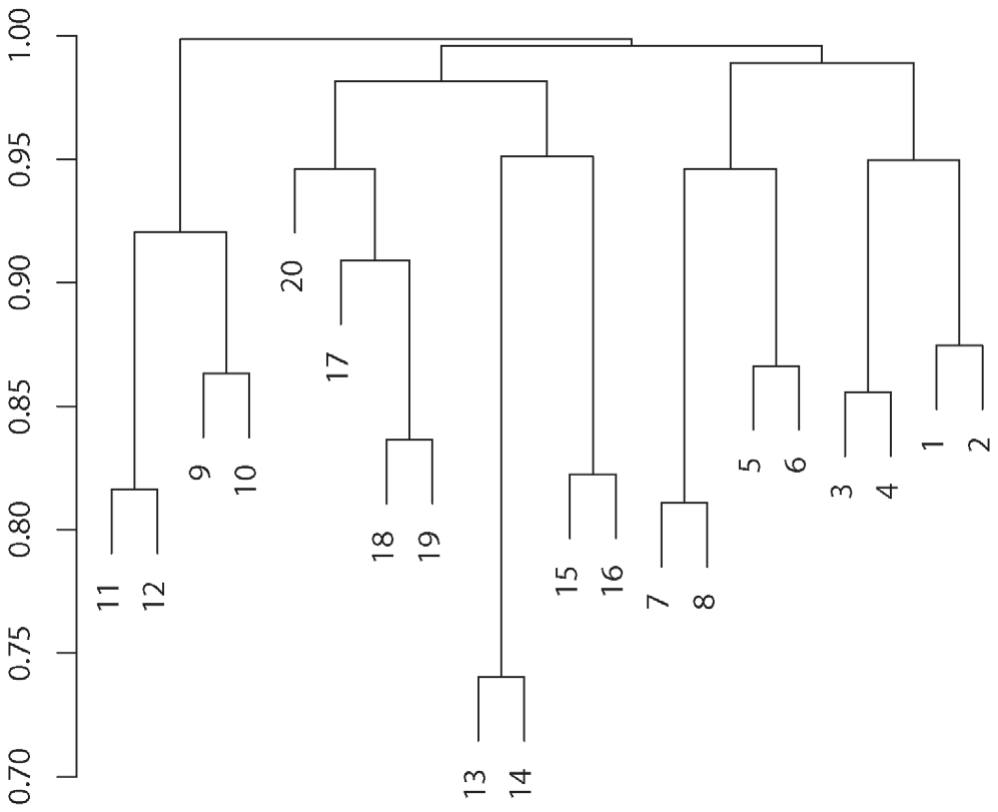
Hierarchical clustering: a dendrogram view showing hierarchical clustering of the genome data generated in the simulation based on the average-linkage method. The resulting grouping recovers the correlation structure in the simulation and leads to the optimal blocking scheme.

Blocking option and blocking parameter (size or number of blocks) unquestionably play an important role in the implementations of *rbPDT* and *rbFBAT*. The optimal choice of blocking depends on mechanisms that are unknown in practice. Our empirical results indicate that gene length, number of SNVs within a gene, and density of SNVs are among factors that may affect the performance of blocking-based methods. As such, we believe that blocking selection should be done in a data adaptive way. For a small to moderately sized gene containing a small to moderate number of tightly scattered SNVs, options with a small number of blocks should be considered. For a large-size gene including many SNVs or moderately large gene with sparsely located SNVs, a large number of blocks will inflate the degrees of freedom whereas a small number of blocks might lead to blocks with distant SNVs. In such cases, selecting the right number of blocks is the key. Another idea in real data analysis (where the underlying truth is unknown) is to use a hierarchical clustering method based on data to partition the genomic region into blocks if the sample size is large and LD within block is strong. We illustrated this by considering a data set with 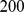 families (GAW 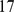 family structure is replicated 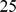 times) simulated in the same way as in our validity study with correlation between adjacent SNVs set to be 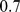. The output of the average-linkage clustering on the genome data is displayed graphically in Figure 6, conveying the existence of five clusters of SNVs 1–4, 5–8, 9–12, 13–16, and 17–20, recovering the grouping scheme in the simulation. If computational intensity in a genome-wide study prevents the selection of a blocking scheme in a data adaptive way, that is automation is desired for practical purpose, then options with a fixed number of blocks may be considered since that would allow an investigator to control the number of blocks.

Another important feature of the proposed methodology is its suitability for detecting association when a mixture of protective and risk variants are involved. The proposed methodology squares the SNV scores before adding them up to prevent cancelation of opposite effects within a block. The advantage of this feature is clearly seen in our simulation results displayed in Table 1. That is *rbPDT* and *rbFBAT* outperform *fbatv0* and *fbatv1*, both of which are susceptible to different directional effects.

There are still many challenges in understanding the involvement of rare variants in complex disease etiology. Given the limited availability and underdevelopment of statistical methods for analyzing family data for detecting rare variant association, we believe that *rbPDT* and *rbFBAT* provide much needed and viable alternatives. Such methods are important in the hunt for rare variant association given the potential enrichment of rare variants in family data and their insensitivity to population stratification.
